# Analysis and injury paterns of walnut tree falls in central anatolia of turkey

**DOI:** 10.1186/1749-7922-9-42

**Published:** 2014-07-01

**Authors:** Suleyman Ersoy, Bedriye Müge Sonmez, Fevzi Yilmaz, Cemil Kavalci, Derya Ozturk, Ertugrul Altinbilek, Fatih Alagöz, Fatma Cesur, Ali Erdem Yildirim, Ozhan Merzuk Uckun, Tezcan Akin

**Affiliations:** 1 Emergency Department, Ahi Evran Univercity Training and Research Hospital, Kırsehir (40100), Turkey; 2 Emergency Department, Ankara Numune Training and Research Hospital, Talatpaşa Bulvarı, Ankara (06100), Turkey; 3 Emergency Department, Baskent Univecity, Taşkent caddesi, Ankara (06490), Turkey; 4 Emergency Department, Şişli Etfal Training and Research Hospital, Halaskargazi caddesi, İstanbul (34371), Turkey; 5 Neurosurgery Department, Ankara Numune Training and Research Hospital, Talatpaşa Bulvarı, Ankara (06100), Turkey; 6 General Surgery Department, Ankara Numune Training and Research Hospital, Talatpaşa Bulvarı, Ankara (06100), Turkey

**Keywords:** Emergency, Falls, Walnut

## Abstract

**Introduction:**

Falls are the second most common cause of injury-associated mortality worldwide. This study aimed to analysis the injuries caused by falls from walnut tree and assess their mortality and morbidity risk.

**Methods:**

This is a retrospective hospital-based study of patients presenting to emergency department (ED) of Ahi Evran Univercity between September and October 2012. For each casualty, we computed the ISS (defined as the sum of the squares of the highest Abbreviated Injury Scale (AIS) score in each of the three most severely injured body regions). Severe injury was defined as ISS ≥ 16. The duration of hospital stay and final outcome were recorded. Statistical comparisons were carried out with Chi-Square test for categorical data and non-parametric spearman correlation tests were used to test the association between variables. A p value less than 0.05 was considered to be statistically significant.

**Results:**

Fifty-four patients admitted to our emergency department with fall from walnut tree. Fifty (92.6%) patients were male. The mean age was 48 ± 14 years. Spinal region (44.4%) and particularly lumbar area (25.9%) sustained the most of the injuries among all body parts. Wedge compression fractures ranked first among all spinal injuries. Extremities injuries were the second most common injury. None of the patients died. Morbidity rate was 9.25%.

**Conclussion:**

Falls from walnut trees are a significant health problem. Preventive measures including education of farmers and agricultural workers and using mechanized methods for harvesting walnut will lead to a dramatic decrease in mortality and morbidity caused by falls from walnut trees.

## Introduction

Falls are the second most common cause of injury-associated mortality worldwide and an important type of blunt trauma which form a significant percentage of traumatic accidents and emergency department admissions [1,2]. Injuries due to falls are largely affected by the height of fall since the velocity and mass of the object determine the kinetic energy which the object gains during fall and is in turn converted to action-reaction forces at the time of impact so as the height increases injury of trauma due to falls becomes more severe although much lesser degree of fall injuries may lead to serious morbidity and mortality [3].

In rural areas where the agriculture is at the forefront, falls from trees constitute a different form of falls from height and as some trees possess unique biological features the severity of injury gains intensity like walnut trees [4,5].

Despite the fact that Turkey is one of the countries considered the homeland of walnut, there is only one study from our country about traumas associated with falls from walnut tree [6] and curiously enough, there were only a few studies in the literature worldwide about this topic (Table 1).

**Table 1:** Details of the studies about falls from walnut tree in literature

	**n**	**Spinal**	**Chest**	**Abdominal**	**Head**	**Extremity**	**Mortality**
		**N (%)**	**N (%)**	**N (%)**	**N (%)**	**N (%)**	**(%)**
Fracture patterns resulting from falls from walnut trees in Kashmir By D.G. Nabi et al.	120	45 (37.5)	1 (0.8)	1 (0.8)	13 (9)	75 (52.9)	
Fall from walnut tree: an occupational hazard by Syed Amin et al.	87	39 (44.8)	21 (24.1)	15 (17.2)	41 (47.1)	23 (26.4)	24.13
Pattern of spine fractures after falling from walnut trees by Seyyed Amirhossein et al.	50	50 (100)					5 (10)
Walnut tree falls as a cause of musculoskeletal injury- a study from a tertiary care center in Kashmir by Asif Nazir et al.	115	52 (45.2)	10 (8.6)	14 (12.1)	34 (29.5)	91 (79)	
Abdominal injury from walnut tree fall. Scientific reports by Imtiaz Wani et al	72	13 (18)	5 (6.9)	17 (23.6)	7 (9.7)	40 (55.5)	5.5
Pattern of trauma related to walnut harvesting and suggested preventive measures by Mudassir M. Wani et al	106	28 (26)	22 (20.7)	8 (7.5)	12 (11.3	90 (84)	5.6

This study aimed to analysis the injuries caused by falls from walnut tree and assess their mortality and morbidity risk.

## Materials and methods

This is a retrospective hospital-based study of patients presenting to emergency department (ED) of Ahi Evran University between September and October 2012. The hospital records of all such patients who were admitted to the ED were studied in detail with regard to patient profile, description and location of the injury, associated injuries, delay in referral, vital signs, labarotory parameters, treatment and survey. For each casualty, we computed the ISS (defined as the sum of the squares of the highest Abbreviated Injury Scale (AIS) score in each of the three most severely injured body regions). Severe injury was defined as ISS ≥ 16. The duration of hospital stay and final outcome were recorded.

All data were analyzed with IBM SPSS software, version 19.0. Results were expressed as mean-standard deviation (SD) or percentage. Statistical comparisons were carried out with Chi-Square test for categorical data and non-parametric spearman correlation tests were used to test the association between variables. A p value less than 0.05 was considered to be statistically significant.

## Results

Falls from walnut trees are a significant health problem owing to being an important source of morbidity and disability from spinal injury, and also a substantial social and economic burden due to labor force loss.

### Demographic data

Fifty-four patients admitted to our emergency department with fall from walnut tree. Of these, 52 were adult and 2 were in pediatric age group. Fifty (92.6%) patients were male and 4 (7.4%) were female. The age range was 14 to 83 years (mean 48 ± 14 years). The earliest admission after the incident occurred at 25th minute and the latest occurred at 24th hour, and the mean delay was 77.96 ± 189.54 minute (Table 2).

**Table 2:** Demographycal and clinical characteristics of patient

**Characteristics**		**n (%)**
Gender	Male	50 (72.6)
	Female	4 (7.4)
Age	Pediatric	2 (3.7)
	Adult	52 (96.3)
Emergency admission time	25 minute (minimum)	
	24 hour (maximum)	
Iinjury severity score (ISS)	1-9	44 (81.5)
	10-15	4 (7.5)
	16-25	9 (11.1)
	25-75	-
Survey	Discharged	19 (35.2)
	Hospitalized	26 (48.1)
	Referred	9 (16.7)
Duration of hospitalization	2 days (minimum)	
	30 days (maximum)	
Clinical outcome	Morbidity (9.25)	
	Mortality (-)	

### Injury patterns

Spinal region (44.4%) and particularly lumbar area (25.9%) sustained the most of the injuries among all body parts. Wedge compression fractures ranked first among all spinal injuries in which 6 were simple of 15 (27.8%) cases. Other types of spinal injuries were as follows: 1 joint dislocation at C3-C4 level, 3 thoracic and 3 lumbar burst fractures, 1 transverse process fracture, and 1 lumbar spinal listhesis. Fourteen patients were exposed to isolated spinal column injuries (SCI), of whom 10 sustained spinal cord injuries leading to 5 paraplegias, 3 paresthesias, 2 quadriparesis, and 1 paraparesis. Neurological complications occurred the most with lumbar region injuries (40%) and with burst fractures (50%). Spinal trauma was most commonly accompanied by cranial injuries (20.8%). Fracture fixation was carried out in 16 patients and 24 patients underwent a conservative management.

Extremities were the second most common (41.7%) injury site after spinal region. Of these, 12 (22.2%) were lower and 10 (18.5%) were upper extremity trauma. While femur and pelvis fractures were the most common injuries among lower extremity traumas, in upper extremity traumas radius fractures were the first (9.3%, 9.3%, and 7.4%, respectively). Eight (36%) of the patients were managed surgically and the other fractures were managed according to the routine orthopedic principles of fracture management. Spinal region injuries, especially the dorsal area, were the most common injuries accompanying both upper and lower extremities (5.3% and 3.1%, respectively).

Fourteen (25.9%) patients had head and neck traumas. No primer traumatic brain injury was observed in any of the patients except for three patients with pneumocephalus. Only 1 patient had a compression fracture in the frontal region and this patient was discharged after a 4-day monitorization period at the neurosurgery department. Spinal injuries were the most common concomitant injury (6.2%).

Eleven (20.4%) patients sustained thoracic trauma and the most common injury specific to this region was rib fractures (16.7%). One patient with multiple rib fractures and hemothorax who underwent tube thoracostomy at the emergency department was operated with urgent thoracotomy as a part of hemorrhagic shock protocol upon drainage of 1300 cc fluid from the chest tube at initial and development of tachycardia (heart rate: 125 bpm) and hypotension (BP: 60/40 mmHg). One patient with pneumomediastinum developed no complication at a 2-week follow-up and was discharged upon regression of the pathology. Yet spinal region injuries were the most common injuries accompanying thoracic injuries (4.9%).

**Figure 1:**
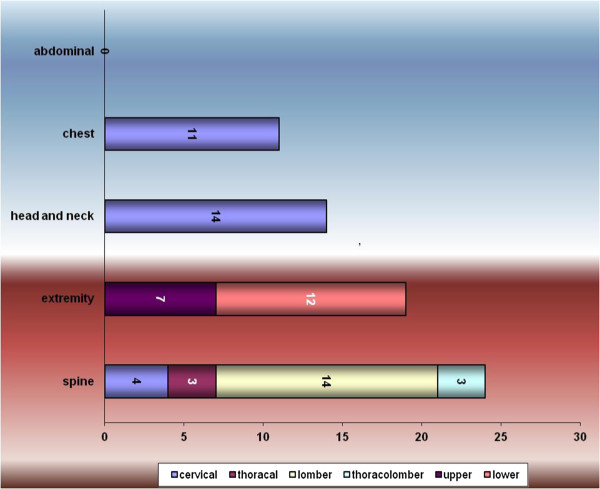
Characteristics of injury paterns.

**Figure 2:**
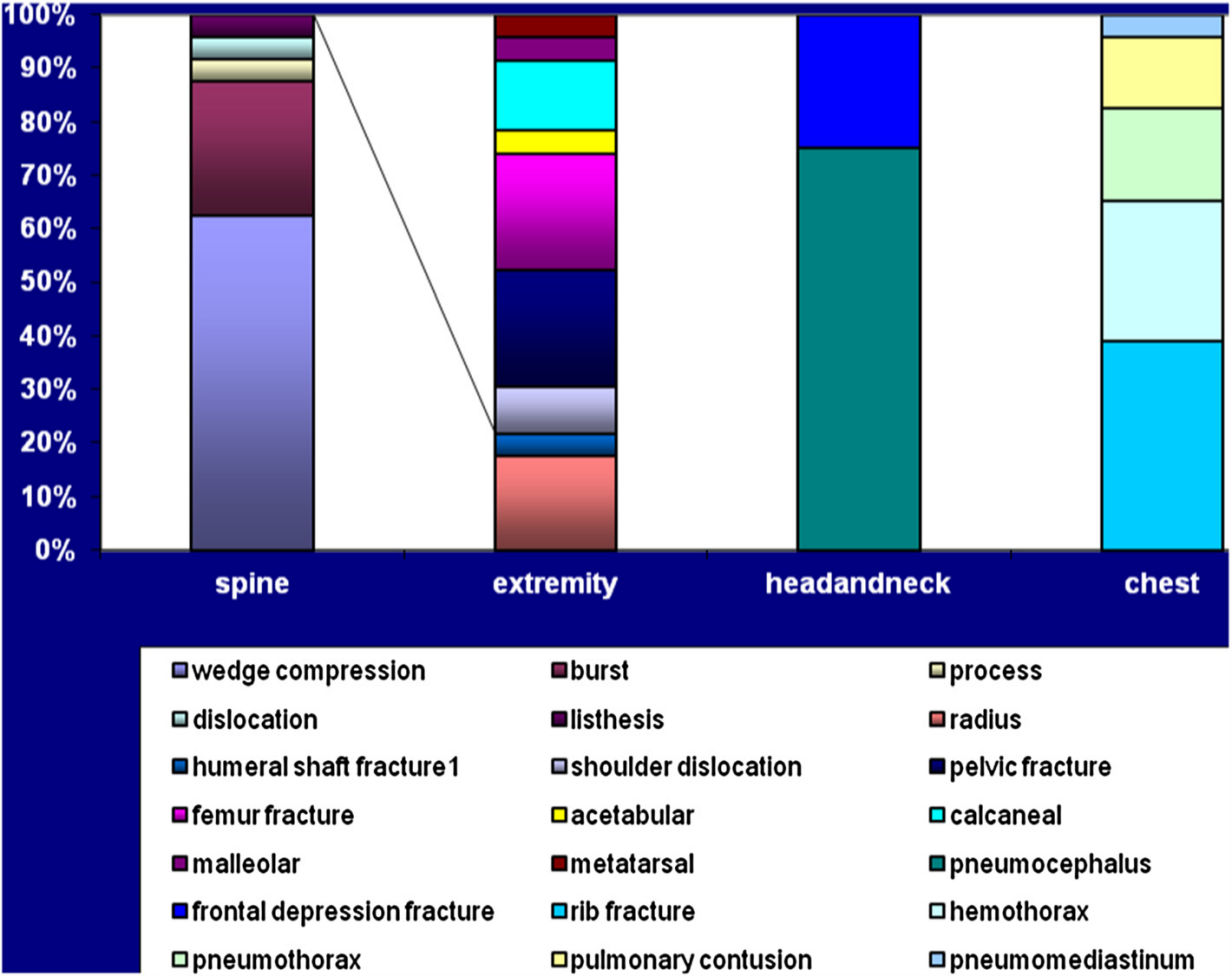
Details of the injury paterns.

Only 1 patient had maxillofacial trauma. Abdominal trauma was not observed in any patient. Thirteen (24%) patients had injuries to more than one anatomical region.Details of the injury paterns were shown on Figures 1 and 2.

### Injury severity score (ISS)

The range of the injury severity score (ISS) was between 1 and 25 (mean 7.4 ± 6 and median 5). Forty-four (81.5%) cases had minor injuries (ISS = 1-9), 4 (7.5%) had moderate injuries (ISS = 10-15), and 9 (11.1%) had severe injuries (ISS = 16-25). There were no critical injuries (ISS = 26-75). The correlation between ISS and duration of hospital stay was strongly positive, linear, and statistically significant (r_s_ = 0.818, p < 0.05). The duration of hospital stay was prolonged as ISS increased (Table 2).

### Survey

Nineteen (35.2%) patients were discharged from emergency department while 26 (48.1%) were hospitalized and 9 (16.7%) were referred to a tertiary center. Department of neurosurgery hospitalized the highest number of patients (33.3%). The mean duration of hospital stay was 6 days (2-30 days) and this duration was ≥10 days in spinal injury patients. Of the hospitalized patients, 14 (40%) were managed surgically and 21 (60%) medically. None of the patients died. Five patients recovered with sequelae and the morbidity rate was 9.25%. Morbidity rate was highest with thoracolumbar injuries (40%) and with burst fractures (40%) (Table 2).

## Discussion

Walnut tree is a species with a great economic importance. The fruit of the walnut tree is used both in food and drug industry, its wood is widely used in furniture sector, and its leaves and roots are utilized in dye manufacturing [7]. The province of Kırşehir located in the Central Anatolian Region and one of its counties, Kaman, has a reputation for its walnut [8].

Although walnut has a great importance in terms of national economy in countries like China, USA, Iran, Turkey and India walnut tree has some unfavorable properties for climbers, including a slippery surface, a substantially tall shaft with a maximum height of 15-30 m and the nuts largely cumulated to distal parts of its branches which are franagible due to the hollow structure [4,9-11]. As falls from heights exceeding 15 meters are accepted high-energy traumas walnut tree falls may result potentially severe injuries [12].

Despite the fact of harvesting walnut by walnut tree machine which shakes the branches of the walnut and eliminate the need to climb the tree, the people of our region continue to harvest walnut by climbing the tree. Falls occur due to the slipping during climbing the tree or while kicking the branches with their foot which breaks them or slipping their feet.

Literature data suggest that males more commonly suffered falls from walnut trees [5,9,13,14]. Our study similarly demonstrated that males more commonly were subjected to injuries (92.6%). The reason of this gender predilection is that the task of walnut harvesting is traditionally fulfilled by males. The injury rate (29.8%) was highest between 51-60 years of age. This has probably stemmed from the fact that the majority of the young population living in this region studied in non-agricultural occupations and choose to live in cities than rural areas.

Patients who fall from walnut tree commonly suffer spine injuries particularly in the form of burst and compression wedge fractures. Spinal injuries have a more destructive influence on clinical outcomes, long-term disability and life quality of patient among all major organ systems although they have a less frequency in trauma victims and especially compression fractures are frequently associated with neurological sequela with increased mortality and long-term morbidity rates [9,14,15]. Our study also demonstrated that the injuries most commonly occurred in the spinal region (44.4%) and wedge compression fractures were the most common spinal injuries (27.8%). Our results were consistent with the literature [9,14] and supports the fact of walnut tree related falls have a serious potential morbidity due to spinal injuries.

Cervical spine injuries at the level of C3-C4 are uncommon, associated bony fractures are infrequent and early agressive management of this level injuries maintain a more favorable outcome in terms of neurological complications [16]. Despite the literature, in the study by Seyed et al. [13] fractures were accompanied dislocations at the cervical level spinal injuries and entirely responsible from all mortality and the results were consistent with the finding of dislocation and fracture at the level of C3-C4 in our study. Quadriparesis was the concomitant neurological deficit in this patient and despite the surgical stabilization patient recovered with sequelae which puts a large social and economic burden on his quality of life as he was a young 35 years old man.

Extremity and head traumas come second after spinal traumas in injuries due to falls from walnut trees and lower limb fractures were more common than upper limb [2,5,14]. We also observed that extremity injuries were the second most common injuries. Consistent with the literature, lower extremity traumas were more common than upper extremity traumas (22.2% and 18.5%, respectively).

In previous studies the mortality rate associated with falls from walnut trees have ranged between 10% to 24.13%, with the majority being due to cervical injuries but on the other hand, we observed no death in our study and this is possibly due to the absence of abdominal injury and existing a few number of head, thoracic and only one cervical trauma patients unlike the literature [5,10,13,14]. Considering the importance of ISS in showing the trauma severity, observing no deaths is consistent with the higher number of patients, 44 (81.5%), with an ISS score of equal to or less than 9. Of 5 patients with sequelae, 3 had an ISS score equal to or greater than 10 and 2 had an ISS score of 9.

## Conclusion

Falls from walnut trees are a significant health problem owing to being an important source of morbidity and disability so are a substantial social and economic burden due to labor force loss. Traditional outdated methods employed in our region for harvesting walnut trees lead to a higher rate of falls from these trees. Preventive measures including education of farmers and agricultural workers and using mechanized methods for harvesting walnut will lead to a dramatic decrease in mortality and morbidity caused by falls from walnut trees.

### Limitations of study

The limitation of our study is related to its duration. The study data were obtained from injuries that took place only during September to October 2012.

## Competing interests

The authors declare that they have no competing interests.

## Authors’ contributions

SE was the lead investigator, BMS carried out the data analysis and writing the manuscript; FY, CK, DO, EA and FA participated in reviewing the manuscript, FC carried out the data analyses; AEY,OMU and TA participated in reducting the language in English. All authors read and approved the final manuscript
